# Effects of changes in intraoperative management on recovery from anesthesia: a review of practice improvement initiative

**DOI:** 10.1186/s12871-015-0040-x

**Published:** 2015-04-23

**Authors:** Toby N Weingarten, Tammy S Bergan, Bradly J Narr, Darrell R Schroeder, Juraj Sprung

**Affiliations:** 1 Department of Anesthesiology, College of Medicine, Mayo Clinic, 200 First Street, SW, Rochester, MN USA; 2 Division of Surgical Services, Department of Nursing, Mayo Clinic, Rochester, MN USA; 3 Division of Biomedical Statistics and Informatics, Department of Health Sciences Research, Mayo Clinic, Rochester, MN USA

**Keywords:** Anesthesia General, Anesthesia Recovery Period, Anesthesia inhalation, Postoperative nausea and vomiting, Hypoventilation

## Abstract

**Background:**

Our anesthetic practice was hindered by inadequate postanesthesia care unit space resulting in operating room inefficiencies. In response, an anesthetic protocol designed to reduce the duration of postanesthesia stay by decreasing residual anesthetic sedation and postoperative nausea and vomiting (PONV) was introduced. Here the impact of this practice change is analyzed.

**Methods:**

The protocol encouraged desflurane use instead of isoflurane, triple antiemetic prophylaxis, and discouraged midazolam. Records of patients undergoing general anesthesia from calendar-matched epochs were reviewed. Epoch I included a 6-month period prior to implementation of the practice change (October 1, 2009, to March 31, 2010) and Epoch II included 6 months following the practice change (October 1, 2010, to March 31, 2011).

**Results:**

General anesthesia was administered to 2,936 and 3,137 patients during Epochs I and II, respectively. Midazolam decreased from 57.4% to 24.0%, isoflurane from 50.8% to 5.7%, desflurane increased from 25.6% to 77.0%, and antiemetic prophylaxis from 6.5% to 50.8%. Median [IQR] recovery time decreased from 72 [50, 102] to 62 [44, 90] minutes, P <0.001. Supplemental analyses found antiemetic prophylaxis was associated with PONV reduction (OR = 0.47, 95% CI 0.38 –0.58, P < 0.001). When compared to isoflurane, desflurane was associated with a decreased rate of respiratory depression (OR = 0.72, 95% CI 0.55-0.93, P = 0.013). Patients administered midazolam trended towards higher rate of respiratory depression (OR = 1.27, 95% CI 1.00–1.60, P = 0.050).

**Conclusions:**

Introduction of an anesthetic protocol that was designed to attenuate adverse anesthetic effects was associated with a reduction of anesthetic recovery time.

## Background

Efficient surgical practices rely on interaction between perioperative and postoperative care areas to facilitate patient throughput [1]. Postoperative care is complex and comprised of multiple clinical areas. The lynchpin of this system is the Postanesthesia Care Unit (PACU) where patients undergo immediate recovery from anesthesia (Phase I recovery) prior to discharge to ambulatory settings, postoperative wards, and advanced monitoring wards (Phase II recovery). When patient volume surpasses PACU capacity, a bottleneck of patient flow is created delaying discharge from the operating room [2]. Slow anesthetic emergence, excessive respiratory depression, and postoperative nausea and vomiting (PONV) can prolong PACU stays [2-4].

Our practice in year 2009 almost daily outstripped PACU capacity which resulted in patient transfer delays from the operating room to PACU. In response, a practice improvement initiative for adult patients undergoing general endotracheal anesthesia (GETA) designed to facilitate Phase I recovery was formulated. This protocol consisted of elements designed to reduce time to emergence from anesthesia and occurrence of respiratory depression (reducing routine midazolam administration, substituting desflurane for isoflurane as the primary inhalational anesthetic) and measures to reduce PONV (triple antiemetic prophylaxis regardless of PONV risk). The primary hypothesis of this study was that this practice change was associated with faster Phase I recovery.

## Methods

This study was approved by the Mayo Clinic, Rochester MN, Institutional Review Board (ID number 13–000171, approved February 5, 2013). Consistent with Minnesota Statute 144.295, all patients provided authorization for research use of their medical records.

### Study design

On August 1, 2010, an anesthesia protocol designed to hasten Phase I anesthesia recovery of patients undergoing GETA was instituted. To assess whether recovery shortened, a retrospective analysis of clinical outcomes before and after protocol implementation was performed. To allow time for acceptance of the protocol a 2-month transition period from protocol institution to the start of data collection was allowed. Therefore, data were obtained during the 6 month period from October 1, 2010, through March 31, 2011 [Epoch II]). In order to ensure that similar calendar periods were compared pre-implementation data were obtained from October 1, 2009, through March 31, 2010 [Epoch I].

### Patient selection

Included were adult patients who underwent GETA, transferred to the PACU, and extubated prior to PACU discharge. Patients were excluded if they bypassed the PACU; had surgery when PACU staffing was not standard (i.e., weekends); or had surgery performed under monitored anesthesia care or regional anesthesia.

### Study setting

This study was of the practice of a *single anesthesia division* within a large anesthesia department. This division provided care for 27 operating rooms which typically serve general, urological, plastic, otolaryngologic, and ophthalmologic specialties as well as endoscopic procedures too complex to be performed in the gastrointestinal procedural suites. Following surgery, patients were transferred to PACU.

### Anesthesia

#### Pre-implementation practice

The anesthesia practice was conducted according to the attending anesthesiologist’s discretion, but typically consisted of an intravenous induction with midazolam, fentanyl, and propofol; maintenance with isoflurane; and antiemetic prophylaxis with ondansetron with or without dexamethasone.

#### Practice improvement protocol

The anesthesia protocol consisted of three practice changes. Two were designed to hasten anesthetic recovery (midazolam was no longer routinely used with induction and desflurane became the default volatile anesthetic) while the third change was aimed to reduce PONV (by using 0.625 mg droperidol, and 4 mg of dexamethasone at the beginning of anesthesia, and 4 mg of ondansetron at the end of anesthesia). Because of the heterogeneity of this practice, there were no recommendations regarding the analgesic regimen. Compliance was not mandatory and anesthesiologists could deviate for individual circumstances.

### PACU clinical practice

The PACU in the clinical practice serves this division as well as other clinical areas (i.e., thoracic, vascular, orthopedic, spine, neurosurgery, and radiology performed under general anesthesia). The PACU does not accept pediatric outpatients nor does it serve as an overflow for the intensive care unit. The PACU is staffed by registered nurses as well as an anesthesia resident. The attending anesthesiologist was also immediately available.

Discharge criteria for Phase I recovery were primarily based on standard discharge criteria, goal pain scores and control of postoperative nausea, as well as for respiratory depression as defined by four *respiratory specific events*, see Table 1 [5-8].

**Table 1:** **Discharge criteria for Phase I recovery following general anesthesia**

Primary Discharge Criteria* [5]	Points		
	0	1	2
Motor activity	No motion	Weak motion	Active motion
Respiration	Required airway maintenance	Maintains airway without support	Coughs on command
Blood pressure	Systolic blood pressure ≥ ± 50 mmHg preanesthetic value	Systolic blood pressure ± 20–50 mmHg preanesthetic value	Systolic blood pressure ± 20 mmHg preanesthetic value
Consciousness	No response or absent protective reflexes	Responds to stimulus	Fully awake or easily aroused
Oxyhemoglobin saturation	<93% or preoperative value with supplemental oxygen	≥93% or preoperative value with supplemental oxygen	≥93% or preoperative value without supplemental oxygen
**Respiratory Specific Events [** 7 **,** 8 **]** ^**†**^			
Hypoventilation	3 episodes of < 8 respirations/minute		
Apnea	Episode of apnea ≥ 10 seconds		
Hypoxemia	3 episodes of oxyhemoglobin desaturations as measured by pulse oximetry (<90% with or without nasal cannula)		
Pain/sedation mismatch	Richmond Agitation Sedation Score[6] = −3 to −5 and a numeric pain score > 5, from a scale 0 to 10		
**Additional Discharge Criteria**			
Numeric Pain Score	Score ≤ 4		
Postoperative nausea	Mild to none		

*To meet discharge criteria the composite score needs to be ≥ 8 with absence of 0 score in any of the 5 subcategories ^†^Any patient who develops a *respiratory specific event* must have a subsequent 60-minute period free of further events in order to be transferred to a nonmonitored ward. Patients who had repeated *respiratory specific events* are discharged to an advanced monitored setting or continuously monitored for oxyhemoglobin desaturation *via* pulse oximetry.

### Data abstraction

Electronic medical records were abstracted using proprietary software [9,10]. Presurgical variables included patient age, sex, body mass index, and American Society of Anesthesiologist Physical Status. Perioperative variables included procedure type; surgical duration; medications; use of regional technique for postoperative analgesia; and Phase I recovery course including duration, medications, and respiratory depression [7,8].

Perioperative dysrhythmia was defined as the use of antiarrhythmic agent or cardioversion, hypertension by the administration of antihypertensive agents, and bronchospasm by albuterol administration. Intraoperative hypotension was assessed from the records of administration of epinephrine, dopamine, calcium chloride, vasopressin, or phenylephrine infusion. Hypotension during Phase I recovery was assessed from the administration of ephedrine or phenylephrine. Antiemetic prophylaxis was determined from the administration of droperidol, dexamethasone, ondansetron or granisetron. PONV was identified from the use of rescue antiemetic medication in the PACU. Perioperative opioids were converted to intravenous morphine equivalents using published guidelines [11,12]. The ultrashort acting remifentanil was not included in morphine equivalent calculations.

The duration of Phase I recovery was defined as the time of PACU admission to the time that Phase I discharge criteria was met. This time was not affected by nonclinical delays in patient transfer from the PACU to Phase II recovery (i.e., patient transport or postsurgical bed availability) [13].

### Statistical analysis

Data are presented as mean ± standard deviation or median [25%, 75%] for continuous variables, and number (percentage) for categorical variables. The primary endpoint was a Phase I recovery time, with secondary endpoint being the rate of PONV, and respiratory specific events. Outcomes were compared between epochs using the rank sum test for continuous variables and the chi square test for categorical variables. Postoperative events which could prolong anesthesia recovery (e.g., respiratory depression, PONV, hemodynamic instability, or increased opioid analgesic administration) were characterized with descriptive statistics. Because this study analyzed a complex practice change, a series of hypothesis-generating secondary analyses *were* performed using multivariable logistic regression to examine the association of the three protocol elements with postoperative PONV and respiratory depression. Two-tailed P values less than 0.05 were considered statistically significant. Statistical analyses were performed with JMP Pro 9.0.1. (SAS Software, Inc., Cary, NC, USA).

## Results

**Figure 1:**
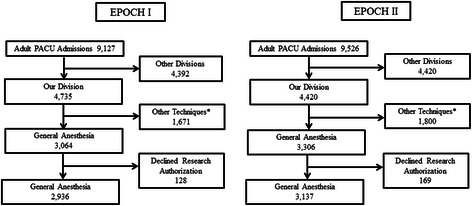
Postanesthesia Care Unit (PACU) population in studied hospital. Legend: *Other techniques include primary regional anesthetics, monitored anesthesia care, general anesthesia with the use of laryngeal mask airway, etc.

**Figure 2:**
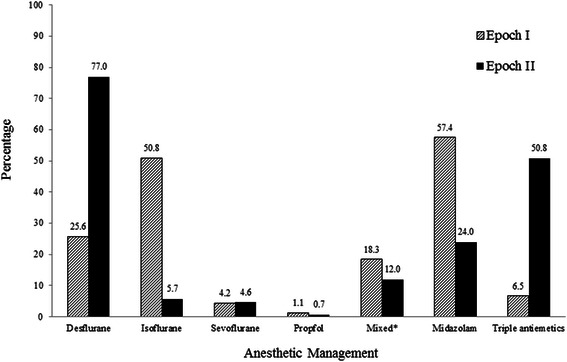
Anesthetic management during two Epochs. Legend: *Mixed anesthetic management included patients who had multiple anesthetic.

General endotracheal anesthesia was administered to 2,936 and 3,137 patients during Epochs I and II, respectively. Figure 1 shows the contribution of these patients to the overall PACU population. Patient, surgical and anesthetic characteristics are presented in Table 2. Changes in anesthetic management between epochs are summarized in Figure 2. Midazolam use decreased 57.4% to 24.0%, desflurane increased from 25.6% to 77.0%, isoflurane decreased from 50.8% to 5.7%, and triple antiemetic prophylaxis increased from 6.5% to 50.8% in Epoch II.

**Table 2:** **Demographics, surgical and anesthetic characteristics**

	Epoch I*	Epoch II*	P
	N = 2,936	N = 3,137	
Age, years	54.8 ± 16.8	54.9 ± 16.8	0.765
Male sex	1,539 (52.5)	1,689 (53.8)	0.291
**ASA-PS**			0.008
I	267 (9.1)	313 (10.0)	
II	1,686 (57.4)	1,731 (55.2)	
III	945 (32.2)	1,019 (32.5)	
IV	38 (1.3)	74 (2.4)	
BMI, kg/m^2^	29.7 ± 7.7	29.4 ± 7.6	0.234
**Surgical type**			0.054
General	1,070 (36.4)	1,034 (33.0)	
Head/Neck	674 (23.0)	811 (25.8)	
Urology	613 (20.9)	642 (20.5)	
Ophthalmology	180 (6.1)	174 (5.5)	
Plastics	168 (5.7)	205 (6.5)	
Gastrointestinal	157 (5.4)	190 (6.1)	
Orthopedics	50 (1.7)	53 (1.7)	
Neurosurgical	13 (0.4)	12 (0.4)	
Thoracic	11 (0.4)	16 (0.5)	
Surgery duration, minutes	129 ± 97	125 ± 93.1	0.131
Intraoperative opioids, iv ME, mg	25 [10,35]	25 [15,35]	<0.001
Intraoperative ketorolac	407 (13.9)	516 (16.5)	0.005
NDMR use^†^	1,594 (54.3)	1,848 (58.9)	<0.001
Neuraxial analgesia used	88 (3.0)	119 (3.8)	0.090
Intraoperative use:			
Bronchodilators	41 (1.4)	64 (2.0)	0.061
Antihypertensives	442 (15.1)	481 (15.3)	0.775
Antiarrhythmics	7 (0.2)	4 (0.1)	0.775
Vasopressors	41 (1.4)	64 (2.0)	0.061

*All patients underwent surgery/procedures under general anesthesia and few had supplemental neuraxial analgesia. ^**†**^NDMR was reversed with neostigmine 1,500 (94.1%) cases during Epoch I and 1,724 (93.3%) during Epoch II where NDMR were used, P = 0.673. Data presented as mean ± standard deviation; number (percentage), or median [25%,75%]. Abbreviations: ASA = American Society of Anesthesiologists Physical Status; BMI = body mass index; iv ME = intravenous morphine equivalents; NDMR = nondepolarizing muscle relaxant

Phase I recovery time decreased by 13.9% (72 [50, 102] vs. 62 [44, 90] minutes in Epoch I and II, respectively, P <0.001) (Table 3). The rates of PONV, respiratory specific events, and administration of antihypertensive medications declined (Table 3). Supplemental analyses found triple antiemetic prophylaxis was associated with PONV reduction (odds ratio 0.47, 95% CI 0.38 – 0.58, P < 0.001). When compared to isoflurane, desflurane was associated with a decreased rate of respiratory depression (odds ratio 0.72, 95% CI 0.55-0.93, P = 0.013). Midazolam use trended towards association to higher rates of respiratory depression (odds ratio 1.27, 95% CI 1.00 – 1.60, P =0.050).

**Table 3:** Duration of Phase I recovery from general anesthesia and clinical outcomes

	Epoch I	Epoch II	P
	N = 2,936	N = 3,137	
Phase I, minutes	72 [50,102]	62 [44,90]	<0.001
PONV requiring treatment	399 (13.6)	261 (8.3)	<0.001
Respiratory events*	229 (7.8)	161 (5.1)	<0.001
Apnea	76	45	
Hypoventilation	107	82	
Oxyhemoglobin desaturation	85	63	
Pain/sedation mismatch	74	48	
Bronchospasm	17 (0.6)	30 (1.0)	0.107
**PACU medications**			
Antihypertensives	266 (9.1)	188 (6.0)	<0.001
Antiarrhythmic medication	7 (0.2)	3 (0.1)	0.213
Vasoactive medication	40 (1.4)	37 (1.2)	0.567
Opioids medication^†^	1.5 [0,10]	0 [0,9]	0.011

*The rate of respiratory events among patients administered nondepolarizing muscle relaxant medications did not differ between patients who were subsequently reversed with neostigmine (204 of 3,180 patients [7.7%]) or were not reversed (14 of 218 patients [6.4%]), P = 0.597. ^†^When excluding patients who did not receive opioids in the PACU, the dose of opioid between epochs did not differ (10 [5,15] iv ME mg *vs.* 10 [5,15] iv ME mg, P = 0.253. Data presented as number (percentage) or median [25%, 75%]. Abbreviations: PONV = postoperative nausea or vomiting; iv ME = intravenous morphine equivalents.

## Discussion

The main finding is that introduction of a protocol designed to reduce the rate of residual anesthetic effects was associated with faster Phase I recovery. Specifically, there was a reduction of oversedation as evidenced by fewer episodes of respiratory depression, and reduction in PONV as evidenced by fewer administrations of antiemetics. Secondary analyses support the notion that the use of desflurane coupled with the avoidance of midazolam was associated with reduced oversedation while antiemetic prophylaxis reduced PONV, and all these effects may have contributed to shorter PACU stay.

Desflurane has a rapid decrease in alveolar concentration after cessation, and in that regard is superior to isoflurane during anesthetic recovery [14-19]. Faster recovery with desflurane over isoflurane have been observed in morbidly obese [19] and elderly patients [17,18], a substantial fraction of the surgical population. While one concern with desflurane has been airway irritability [20], albuterol use did not differ between epochs, suggesting there was not increases of bronchospasm. However, selection bias for sevoflurane in patients with reactive airway disease cannot be excluded.

Because midazolam is associated with increased Phase I recovery, the protocol narrowed its indication to patients undergoing invasive awake procedures or experiencing notable anxiety. The effects of midazolam on Phase I recovery have not been extensively studied. One prospective study of 90 elderly patients undergoing transurethral procedures under desflurane anesthesia found midazolam prolonged PACU discharge time and increased incidence of oxyhemoglobin desaturations [21]. Another prospective study of 30 women undergoing laparoscopic tubal sterilization under nitrous oxide and isoflurane found increase sedation during Phase I recovery [22]. Another prospective study of 88 nonobese adult ambulatory patients found that midazolam did not affect PACU stay [14]. A supplemental analyses found an association between respiratory depression and isoflurane and a trend with midazolam suggesting that both components can adversely impact anesthesia recovery.

Triple antiemetic prophylaxis regimens reduce PONV [23], (an association observed in this study supplemental analyses), which contributes to faster Phase I recovery. No patients in this study who received droperidol experienced adverse cardiac effects (dysrhythmias associated with long QT interval, a concern that triggered FDA to issue a “black box” warning) [24]. A confounding observation is the decreased use of opioids in the PACU during Epoch II which may be explained by the modest increase of intraoperative opioid administration. This decline in administration could have contributed to the decline in PONV and respiratory depression. Another unexplained observation was decreased use of antihypertensives in Epoch II.

### Limitations

This study has the inherent limitations of a retrospective study design. Though the anesthesia protocol in Epoch II was widely adopted, it was not universally so. Reasons for variance may include residual practice bias and clinical factors which could introduce a treatment bias where anesthetic technique could be altered to account for specific patient risk factors. Although the formal practice change was implemented on August 1, 2010, informal adoption of protocol components may have occurred prior to that date. Because the practice protocol was multifaceted assessing the impact of individual components is difficult, but a series of hypothesis generating supplemental analyses support the speculation that individual components contributed to clinical improvements. However, other factors could contribute to clinical outcomes such as inadequate reversal of neuromuscular blocking drugs and respiratory depression. Unaccounted management changes could have impacted PACU efficiency; however no changes in staffing or discharge protocol were made during the study timeframe. Finally, though the Phase I recovery audit was performed retrospectively, we cannot exclude a potential Hawthorne effect by healthcare staff in anticipation of practice evaluation following protocol implementation.

## Conclusions

The introduction of an anesthetic protocol that aimed to reduce adverse effects of anesthetics was associated with a reduction of Phase I recovery time in adult patients undergoing general endotracheal anesthesia. These anesthetic management changes were primarily associated with decreased rate of postoperative respiratory depression and nausea and vomiting.

## Abbreviations

PONV: Postoperative nausea and vomiting
PACU: Postanesthesia Care Unit
GETA: General endotracheal anesthesia
BMI: Body mass index
